# Pulsed Electric Field Inactivation of *Acetobacter aceti*: Mechanisms and Efficacy Elucidation

**DOI:** 10.3390/foods14244188

**Published:** 2025-12-06

**Authors:** Yongxin Teng, Boru Chen, Runyu Yao, Langhong Wang, Zhong Han, Xin-An Zeng

**Affiliations:** 1 Guangdong Key Laboratory of Intelligent Food Manufacturing, Foshan University, Foshan 528225, China; mstengyongxin1997@mail.scut.edu.cn (Y.T.); wlhong@fosu.edu.cn (L.W.); 2 School of Food Science and Engineering, South China University of Technology, Guangzhou 510641, China; 202110185808@scut.edu.cn (R.Y.); fezhonghan@scut.edu.cn (Z.H.)

**Keywords:** wine acetification, *Acetobacter aceti*, pulsed electric field, membrane damage, enzyme inactivation, electroporation

## Abstract

The spoilage bacterium *Acetobacter aceti* poses a major threat to wine quality by causing acetification, driving the need for effective control methods. This study investigated the inactivation of *A. aceti* using pulsed electric field (PEF) and elucidated the multi-target mechanisms. The results demonstrated that PEF efficacy was highly dependent on the electric field intensity. PEF treatment at 30 kV/cm achieved a >3-log reduction in viable cell counts, with a Weibull model analysis indicating a critical inactivation threshold of 21.64 kV/cm. Mechanistic investigations revealed that PEF induced irreversible damage to the cell membrane, evidenced by morphological rupture (SEM) and a 4-fold increased permeability (flow cytometry), which led to a massive leakage of intracellular contents. Critically, this physical damage was correlated with profound physiological disruption, including the inactivation of key spoilage enzymes alcohol dehydrogenase (ADH, 80.0% relative activity loss) and aldehyde dehydrogenase (ALDH, 93.1% relative activity loss). Furthermore, PEF induced severe oxidative stress (4.25-fold increase in ROS and 3.30-fold increase in MDA) and a collapse in energy metabolism. Collectively, these findings reveal a synergistic inactivation mechanism, which establishes a strong theoretical foundation for the potential development of PEF as a non-thermal strategy to control acetic spoilage in winemaking.

## 1. Introduction

Wine is a globally cherished alcoholic beverage with significant economic and cultural value, primarily produced through the alcoholic fermentation of grape juice by yeasts [1]. However, the winemaking process involves a complex microbial ecosystem, encompassing not only the fermentative yeasts but also a diverse array of indigenous microorganisms originating from the grapes, winery equipment, and environment [2]. The proliferation of spoilage microorganisms is particularly pronounced when grapes are of suboptimal quality, such as being damaged or rotten, which leads to a higher initial microbial load. Among these undesirable microbes, acetic acid bacteria (AAB), particularly species within the *Acetobacter* genus such as *A. aceti*, pose a significant threat to wine quality [3]. These strictly aerobic bacteria can rapidly form a pellicle on the wine surface upon exposure to air, metabolizing ethanol into acetic acid [4,5]. This process, known as acetic spoilage, results in a sharp increase in volatile acidity, alongside turbidity and the development of undesirable off-flavors, leading to severe quality degradation and substantial economic losses for the wine industry.

Conventionally, the primary strategy for controlling microbial growth throughout the winemaking process has been the application of sulfur dioxide (SO_2_) [6]. Rather than being added as a gas, SO_2_ is typically introduced in the form of sulfite salts, most commonly potassium metabisulfite (K_2_S_2_O_5_), which acts as a versatile antimicrobial and antioxidant agent from grape crushing to final bottling [7]. Additionally, small amounts of SO_2_ are naturally produced by yeast during alcoholic fermentation [8]. However, the reliance on SO_2_ is carefully managed due to significant drawbacks. From a sensory perspective, excessive concentrations can impart a pungent, unpleasant aroma to the wine [9]. More critically, SO_2_ is a known allergen for a subset of the population, capable of triggering allergic-type reactions. This health concern has led to strict regulatory controls and mandatory “Contains Sulfites” labeling in most countries if the concentration exceeds 10 mg/L [10]. For instance, the European Union, a major regulatory body, restricts total SO_2_ content to 150 mg/L for most red wines and 200 mg/L for white and rosé wines [9]. Consequently, driven by a growing consumer demand for “clean label,” “natural,” and “low-/no-sulfite” wines, there is a pressing need for the development of novel, green, and effective strategies for microbial control.

Currently, non-thermal processing technologies have garnered considerable attention as promising alternatives to conventional pasteurization for heat-sensitive liquid foods such as fruit juices, milk, and wine [11,12]. These technologies, including high-pressure processing (HPP), cold plasma, ultrasound, and pulsed electric field (PEF), can inactivate spoilage microorganisms while minimizing adverse effects on the product’s nutritional and sensory properties [13]. Among these, PEF technology is considered one of the most viable for industrial applications due to its significant advantages, e.g., short treatment times, suitability for continuous operation, and uniform treatment effects [14]. The microbial inactivation efficacy of PEF has been widely demonstrated across various food matrices [15]. For instance, a PEF treatment at 35 kV/cm reduced total aerobic bacteria and fungi in strawberry juice by 1.21 and 1.60 log CFU/mL, respectively, while a 34 kV/cm treatment achieved over a 5-log reduction in *Escherichia coli* and *Salmonella Typhimurium* in vegetable juice [16,17]. However, the effectiveness of PEF is highly dependent on the processing parameters. For example, while PEF at 20 kV/cm resulted in significant reductions in *E. coli* (>8 log) and *Listeria innocua* (>4 log) in a mixed juice, its efficacy dropped to less than a 1-log reduction when the field strength was lowered to 10 kV/cm [18]. Similarly, Astrid et al. reported that a low field strength of 6 kV/cm was insufficient to cause significant inactivation of *E. coli* [19]. These findings collectively highlight that achieving desirable inactivation efficiency often requires carefully optimized parameters, particularly a sufficiently high electric field strength.

The application of PEF for inactivating wine-related spoilage microorganisms has also been documented [20,21]. Several studies have demonstrated its effectiveness against various spoilage yeasts (e.g., *Brettanomyces bruxellensis* [22], the yeast responsible for the undesirable “Brett” off-flavors in red wines) and bacteria (e.g., *Lactobacillus* and *Pediococcus* spp. [23,24], causing sluggish fermentations or unwanted malolactic fermentation) in a simulated wine environment. Some studies have even advanced to treating real grape must and juice, showing that PEF can reduce the native microbial load before fermentation, effectively acting as a “pre-treatment” step [25,26]. However, research focusing specifically on the PEF inactivation of *Acetobacter* spp., the primary causative agents of acetic spoilage, remains limited. The few existing studies have primarily investigated the synergistic effects of PEF when combined with other stressors. For instance, Niu et al. examined the enhanced inactivation of *Acetobacter* spp. by combining PEF with different ethanol concentrations, while another study explored the synergy between PEF and resveratrol [27,28]. While valuable, these works have centered on combined treatments, leaving the intrinsic mechanism of PEF as a standalone technology largely unaddressed. Consequently, a comprehensive investigation that moves beyond confirming general principles (e.g., electroporation [29]) to build a complete, multi-target inactivation model—linking primary membrane damage to subsequent metabolic collapse and functional failure specifically for *A. aceti*—is critically needed. This gap in knowledge hinders the rational optimization of PEF for controlling this specific spoilage organism.

To address this knowledge gap, this study was designed to systematically evaluate the inactivation of the wine spoilage bacterium (*Acetobacter aceti*) in a simulated wine environment using PEF as a sole technological intervention. First, the impacts of key PEF processing parameters (electric field strength, pulse frequency, pulse width, and residence time) on the inactivation of *A. aceti* were systematically investigated. Subsequently, with electric field strength identified as a critical variable, a multi-level investigation was conducted to elucidate the underlying inactivation mechanisms, from the cell exterior to its internal functions. Specifically, the investigation focused on the damage to the primary target, the cell membrane, by assessing its integrity, micro-morphology, and lipid composition. Further analyses explored the consequent leakage of intracellular materials (e.g., nucleic acids and proteins) and the disruption of internal cellular structures. Finally, this physical damage was correlated with the bacterium’s core physiological functions by measuring the relative activity of key acid-producing enzymes (alcohol dehydrogenase and aldehyde dehydrogenase) and evaluating the overall impact on cellular energy metabolism and oxidative stress levels. The findings aim to establish a fundamental mechanistic understanding that can serve as a theoretical foundation for the future development and optimization of PEF as a potential non-thermal, sulfite-free technology to control acetic acid spoilage in winemaking.

## 2. Materials and Methods

### 2.1. Materials

The strain *Acetobacter aceti* CGMCC 1.41, previously isolated from spoiled wine in our laboratory, was used in this study. The strain was cryopreserved at −80 °C in Glucose-Peptone-Yeast Extract (GPYA) medium supplemented with 30% (*v*/*v*) glycerol. For activation, the frozen stock was streaked onto GPYA agar plates (glucose 50 g/L, yeast extract 10 g/L, peptone 10 g/L, and agar 20 g/L; pH adjusted to 6.8) and incubated at 30 °C for 48 h. A single colony was then inoculated into 100 mL of GPYA liquid medium and cultivated at 30 °C with shaking at 170 rpm for 48 h to obtain a seed culture. Cells were harvested for experiments during the logarithmic growth phase. Propidium iodide (PI) was purchased from Thermo Fisher Scientific (Waltham, MA, USA). The Supelco^®^ 37 Component FAME Mix was obtained from Merck (Darmstadt, Germany). Commercial assay kits for alcohol dehydrogenase (ADH, Cat. No. BC1085), aldehyde dehydrogenase (ALDH, Cat. No. BC0750), reactive oxygen species (ROS, Cat. No. IKC10621), ATP content (Cat. No. BC0300), and malondialdehyde (MDA, Cat. No. BC0025) were all purchased from Solarbio Science & Technology Co., Ltd. (Beijing, China). All other chemicals and reagents were of analytical grade and procured from Sinopharm Chemical Reagent Co., Ltd. (Shanghai, China). Deionized water was used throughout the experiments.

### 2.2. PEF Treatment

The cell culture was centrifuged at 5000× *g* for 10 min at 4 °C. The supernatant was discarded, and the cell pellet was washed three times with sterile 0.1 M acetate buffer (pH 3.5) to completely remove residual medium. Finally, the cells were resuspended in the same acetate buffer. The final cell concentration was adjusted to approximately 10^6^ CFU/mL, and the electrical conductivity of the suspension was standardized to 500 µS/cm using the same buffer to simulate wine conditions and ensure experimental uniformity.

**Figure A1:**
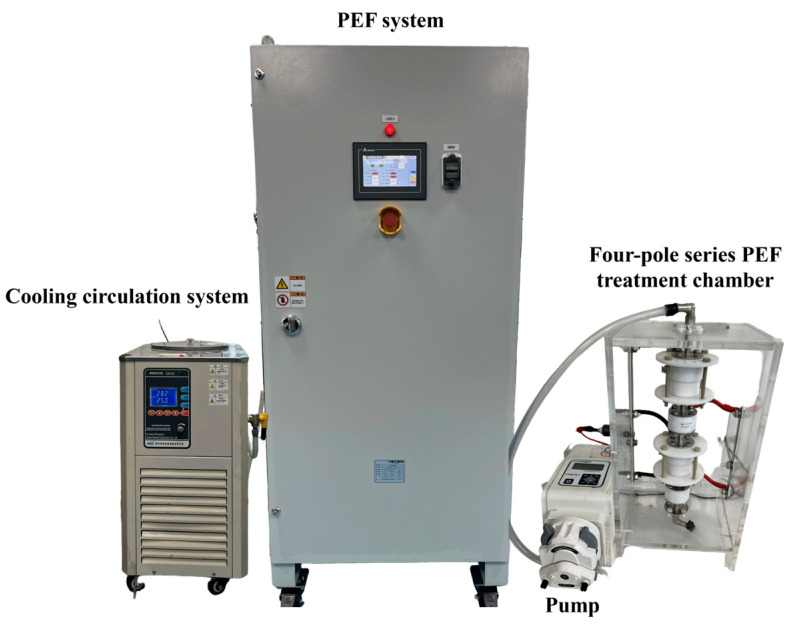
Illustration of pulsed electric field equipment.

PEF treatment was performed using custom-built, experimental-grade PEF equipment (Guangzhou Paihu Technology Co., Ltd., Guangzhou, China), a schematic of which is provided in Appendix A Figure A1. The PEF system utilizes a treatment unit composed of four independent treatment chambers connected in series. Each individual chamber features a coaxial cylindrical electrode configuration, where two cylindrical electrodes are arranged concentrically around the central pipeline through which the sample flows. The electric field is applied radially across the fluid in the treatment gap (0.30 cm), resulting in an effective cross-sectional area of 0.1256 cm^2^ for the treatment. The system generates square-wave pulses. For each treatment, 100 mL of the cell suspension was processed at a continuous flow rate of 100 mL/min. The system was integrated with a heat exchanger, circulating 4 °C water to minimize thermal effects. The temperature of the suspension before and after PEF treatment was monitored, with the increase consistently maintained below 5 °C. The PEF treatment parameters and corresponding specific energy input (Q) were varied as follows:

Electric field intensity (EFI): 10, 15, 20, 25, and 30 kV/cm (fixed frequency: 300 Hz, pulse width: 10 µs, treatment time: 9.0 ms per milliliter of the sample, corresponding processing time is 5 min). The specific energy inputs were 0.68 × 10^2^, 1.53 × 10^2^, 2.71 × 10^2^, 4.24 × 10^2^, 6.10 × 10^2^ kJ/L, respectively.

Pulse frequency (PF): 100, 150, 200, 250, and 300 Hz (fixed EFI: 30 kV/cm, pulse width: 10 µs, treatment time: 9.0 ms per milliliter of the sample, corresponding processing time is 5 min). The specific energy inputs were 2.03 × 10^2^, 3.05 × 10^2^, 4.07 × 10^2^, 5.09 × 10^2^, 6.10 × 10^2^ kJ/L, respectively.

Pulse width (PW): 2, 4, 6, 8, and 10 µs (fixed EFI: 30 kV/cm, frequency: 300 Hz, treatment time: 9.0 ms per milliliter of the sample, corresponding processing time is 5 min). The specific energy inputs were 1.22 × 10^2^, 2.44 × 10^2^, 3.66 × 10^2^, 4.88 × 10^2^, 6.10 × 10^2^ kJ/L, respectively.

Treatment time (RT): 1.8, 3.6, 5.4, 7.2, and 9.0 ms per milliliter of the sample, corresponding processing time is 1, 2, 3, 4, 5 min, respectively, (fixed EFI: 30 kV/cm, frequency: 300 Hz, pulse width: 10 µs). The specific energy inputs were 1.22 × 10^2^, 2.44 × 10^2^, 3.66 × 10^2^, 4.88 × 10^2^, 6.10 × 10^2^ kJ/L, respectively.

The specific energy input was calculated using the following Equations (1) and (2).

(1) $$t\left(s\right)=T\left(\min\right)\times 60\times F(\mathrm{Hz})\times P(\mu s)/\text{1,000,000}$$

(2) $$Q\left(\mathrm{kJ}/L\right)=\frac{E^{2}\times D\times t\times N\times A\times \Phi }{V\times 1000}$$

where t is the effective treatment time (s), T is the processing time (min), F is the pulse frequency (Hz), P is the pulse width (μs); Q is the energy per liter of protein solution processed (kJ/L), E is the electric field intensity (kV/cm), D is the parallel electrodes distance (0.3 cm), N is the treatment chamber number (4), A is the sectional area of treatment chamber (0.1256 cm^2^), Φ is the electrical conductivity (500 µS/cm), V is the volume of protein solution (0.1 L).

Samples not subjected to PEF treatment served as the control group (0 kV/cm). All treatments were performed at room temperature, and the treated samples were immediately placed in an ice bath to prevent thermal effects. Samples were then centrifuged at 5000× *g* for 10 min at 4 °C, and the pellets and supernatants were collected separately for subsequent analyses.

### 2.3. Microbiological Analysis and Inactivation Kinetics

The viability of *A. aceti* was determined by the plate count method. Treated cell suspensions were serially diluted (10^−1^ to 10^−5^) in sterile 0.85% (*w*/*v*) saline solution. Aliquots of 100 µL from appropriate dilutions were spread onto GPYA agar plates in triplicate. The plates were incubated inverted at 30 °C for 48 h. The results were expressed as colony-forming units per milliliter (CFU/mL). The log reduction was calculated using Equation (3):

(3) $${P=Log}_{10}(\frac{N_{1}}{N_{0}})$$

where N_0_ is the number of viable cells in the control group (CFU/mL), and N_1_ is the number of viable cells in the treated group (CFU/mL).

The inactivation kinetics of *A. aceti* under different PEF treatment conditions (EFI, PF, PW, and RT) were described using the Weibull distribution model Equation (4) [29]:

(4) $${Log}_{10}\left(\frac{N_{1}}{N_{0}}\right)=-{\left(\frac{x}{a}\right)}^{b}$$

where x is the value of the PEF parameter (e.g., EFI), a is the scale parameter representing the treatment intensity required for the first decimal reduction, and b is the shape parameter indicating the concavity (b < 1) or convexity (b > 1) of the survival curve. The fitting was performed with the Levenberg–Marquardt iteration algorithm, applying instrumental weighting to account for the variance in the experimental data points. The goodness-of-fit for each model was evaluated based on the coefficient of determination (R^2^ > 0.95) and the reduced chi-square value. The number of iterations was set to a maximum of 400, and convergence was achieved in all cases.

### 2.4. Membrane Fatty Acid Composition Analysis

Fatty acids were converted to fatty acid methyl esters (FAMEs) using a standard saponification-methylation method [30]. Briefly, 40 mg of lyophilized powder was saponified with 1.0 mL of NaOH-methanol solution (15 g NaOH in 50 mL methanol and 50 mL water) at 100 °C for 30 min. After cooling, the samples were methylated with of HCl-methanol solution at 80 °C for 10 min. FAMEs were then extracted with 1.25 mL of a hexane/methyl tert-butyl ether (1:1, *v*/*v*) solution. The organic phase was washed with NaOH solution. The upper organic layer was collected for GC-MS analysis.

GC-MS analysis was performed on an Agilent 8890-7000D system (Agilent Technologies, Santa Clara, CA, USA) equipped with an Agilent HP-INNOWax capillary column (30 m × 0.25 mm × 0.25 µm, Agilent Technologies, Santa Clara, CA, USA). Helium was the carrier gas at a flow rate of 1.0 mL/min. The injector temperature was 250 °C, and the injection volume was 1 µL in splitless mode. The oven temperature program was: 150 °C for 5 min, then ramped at 5 °C/min to 250 °C and held for 5 min. The mass spectrometer was operated in electron impact (EI) mode at 70 eV with an ion source temperature of 230 °C and a quadrupole temperature of 150 °C. The mass scan range was m/z 30–500. Fatty acids were identified by comparing their mass spectra with the NIST library and their retention times with the FAME standard mix. Relative contents were calculated using the peak area normalization method.

### 2.5. Scanning Electron Microscopy

Cell pellets were washed three times with 0.1 M phosphate-buffered saline (PBS, pH 7.2) and fixed overnight in 2.5% (*v*/*v*) glutaraldehyde at 4 °C. After washing with PBS, the samples were dehydrated through a graded ethanol series (30%, 50%, 70%, 80%, 90%, and 100%; 15 min each), followed by two changes in absolute ethanol. The samples were then subjected to tertiary-butyl alcohol replacement and air-dried. The dried samples were sputter-coated with gold for 120 s and observed under a field-emission SEM (Hitachi SU8010, Tokyo, Japan) at an accelerating voltage of 3.0 kV [31].

### 2.6. Flow Cytometry Analysis

Membrane permeability was assessed using PI staining [28]. A 1 mL aliquot of the treated cell suspension was washed and resuspended in 1 mL of PBS. Then, 5 µL of a 1 mg/mL PI solution was added, and the mixture was incubated in the dark at room temperature for 20 min. After washing to remove excess dye, the samples were analyzed using a flow cytometer (FACSAria III, BD Biosciences, San Jose, CA, USA) with an excitation wavelength of 488 nm.

### 2.7. Transmission Electron Microscopy

Cell pellets were pre-fixed with 2.5% glutaraldehyde at 4 °C for 4 h, washed with PBS, and post-fixed with 1% (*w*/*v*) osmium tetroxide at 4 °C for 2 h. After washing with PBS, the samples were dehydrated with a graded ethanol series and acetone. The samples were then infiltrated and embedded in epoxy resin, followed by polymerization at 60 °C for 48 h. Ultrathin sections (60 nm) were prepared using an ultramicrotome (Leica EM UC7, Wetzlar, Germany), mounted on copper grids, and double-stained with 2% uranyl acetate and 0.4% lead citrate. The ultrastructure was observed using a TEM (JEM-1400 Plus, JEOL, Tokyo, Japan) at an accelerating voltage of 80 kV [32].

### 2.8. Measurement of Intracellular Component Leakage

Supernatants from treated samples were collected by centrifugation (8000× *g*, 10 min, 4 °C). The electrical conductivity of the supernatant was measured using a conductivity meter. The leakage of nucleic acids and proteins was quantified by measuring the absorbance of the supernatant at 260 nm and 280 nm, respectively, using a UV-Vis spectrophotometer (Shimadzu UV-2600, Kyoto, Japan), with the acetate buffer as a blank [33].

### 2.9. Determination of Key Physiological Indicators

The relative activities of ADH and ALDH, and the intracellular contents of ROS, MDA, and ATP were determined using their respective commercial assay kits (Solarbio, Beijing, China).

### 2.10. Statistical Analysis

All experiments were performed in triplicate, and data are presented as the mean ± standard deviation (SD). Statistical analysis was conducted using OriginPro 2024 software (OriginLab, Northampton, MA, USA). One-way analysis of variance (ANOVA) followed by Duncan’s multiple range test was used to determine significant differences between groups. A significance level of *p* < 0.05 was adopted for all analyses.

## 3. Results and Discussion

### 3.1. Effect of PEF Processing Parameters on the Inactivation of A. aceti

**Figure 1:**
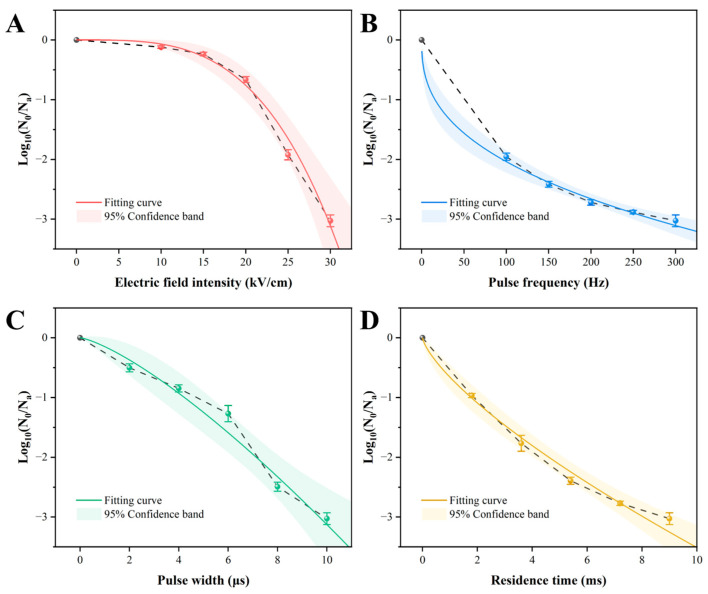
Inactivation curves of *Acetobacter aceti* treated with different PEF parameters, fitted with the Weibull model. The shaded areas represent the 95% confidence intervals. (**A**) Electric field intensity; (**B**) pulse frequency; (**C**) pulse width; (**D**) residence time.

The efficacy of PEF treatment against *Acetobacter aceti* was evaluated by varying four key parameters: electric field intensity (EFI), pulse frequency (PF), pulse width (PW), and residence time (RT). All parameters demonstrated a significant, dose-dependent lethal effect (Figure 1). Notably, EFI exerted the most pronounced impact, exhibiting a clear threshold effect: inactivation was marginal below 15 kV/cm but surged 7-fold (from 0.24 to 1.92 log CFU/mL) as EFI increased from 15 to 25 kV/cm, reaching a 3.03-log reduction at 30 kV/cm (Figure 1A). To quantitatively analyze these dynamics, the experimental data were fitted to the Weibull model, which provided an excellent description (R^2^ > 0.97) for all conditions (Table 1). The model’s scale parameter (a), representing the treatment intensity for a 1-log reduction, highlighted the decisive role of EFI. The high a value for EFI (21.64 kV/cm) compared to the relatively low values for PF, PW, and RT underscores that a substantial electric field strength is a prerequisite for effective inactivation, establishing it as the rate-limiting parameter. This finding aligns with previous studies where EFI was consistently identified as the most critical factor governing PEF lethality [17,34,35]. Furthermore, the low a value for RT (1.77 ms) highlights the potential for rapid processing, a key advantage for treating heat-sensitive liquids like wine [14].

The shape parameter (b) provided further insight into the inactivation mechanism. For EFI, the large b value of 3.48 ± 0.12 corresponds to a distinct convex curve (b > 1, Figure 1A), indicating a rapid collapse of the microbial population’s resistance above a certain intensity. This is highly consistent with the theory of electroporation, where cell death occurs abruptly once the transmembrane potential exceeds a critical threshold, leading to irreversible pore formation [29]. The a value of 21.64 kV/cm can thus be interpreted as the approximate critical field strength needed to trigger this lethal event in *A. aceti*. In contrast, the concave curves observed for RT and PF (b < 1) suggest a “tailing effect,” where inactivation efficiency diminishes over time, possibly due to a resistant subpopulation (Figure 1B,D).

In summary, PEF treatment can achieve over a 3-log reduction in *A. aceti*, with EFI being the critical parameter that governs the process via a threshold-dependent mechanism consistent with electroporation. Based on these findings, EFI was selected as the key variable for subsequent investigations into the cellular and physiological inactivation mechanisms. It is important to note that while 30 kV/cm proved to be a highly effective intensity in our model system, the concept of a single “optimal” EFI is context-dependent. In practice, the optimal value represents a crucial trade-off between achieving the desired level of microbial inactivation, minimizing energy consumption, and preserving the sensory and chemical integrity of the final product. Therefore, while our findings establish 21.64 kV/cm as the critical threshold for initiating cell death and 30 kV/cm as a benchmark for high efficacy, the true optimal parameters for a specific wine application would need to be further explored to balance these complicated factors.

### 3.2. Impact of PEF on the Cell Membrane of A. aceti

The cell membrane is the primary target of PEF-induced electroporation, and its structural and functional integrity is decisive for cell survival [29]. Therefore, to elucidate the inactivation mechanism, this section systematically investigates the damage to the *A. aceti* cell membrane from three perspectives: micro-morphology, physiological integrity, and biochemical composition.

#### 3.2.1. Morphological Damage Visualized by SEM

**Figure 2:**
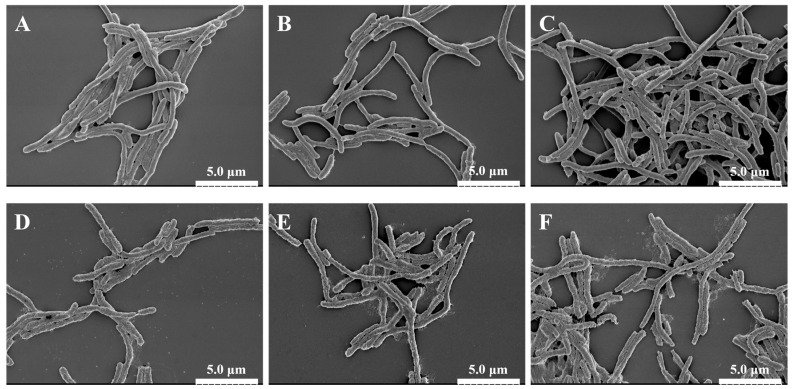
Scanning electron microscopy (SEM) images (×6000 magnification) showing the morphological changes in *A. aceti* cells after PEF treatment at different electric field intensities. (**A**) Control (0 kV/cm); (**B**) 10 kV/cm; (**C**) 15 kV/cm; (**D**) 20 kV/cm; (**E**) 25 kV/cm; (**F**) 30 kV/cm. Scale bar = 5 µm.

Scanning electron microscopy (SEM) was used to directly visualize the damage inflicted on *A. aceti* cells by PEF treatment. As shown in Figure 2, the treatment induced progressive, intensity-dependent morphological alterations. The control cells exhibited a typical smooth, intact, and plump rod shape (Figure 2A). At low EFIs (10–15 kV/cm), the cells showed only minor surface roughening (Figure 2B,C). However, as the EFI increased to 20 kV/cm, significant damage became apparent, with widespread surface wrinkling and cell shrinkage observed (Figure 2D). This level of damage, observed at the previously identified inactivation threshold (Section 3.1), indicates a significant loss of turgor pressure due to compromised membrane permeability [27].

When the EFI surpassed this threshold to 25 and 30 kV/cm, the cellular damage escalated dramatically. In addition to severe wrinkling, clear evidence of surface pitting, pore formation, and even cell lysis into debris was observed (Figure 2E,F). These features provide direct morphological evidence of irreversible electroporation. Similar PEF-induced damage, including pore formation and cell rupture, has been widely reported for other bacteria such as *E. coli* and *S. aureus* at comparable field strengths, confirming that physical disruption of the cell envelope is a universal mechanism of PEF inactivation [31,36]. These results visually confirm that PEF inactivates *A. aceti* by progressively destroying the physical integrity of the cell membrane.

#### 3.2.2. Quantitative Analysis of Membrane Integrity by Flow Cytometry

**Figure 3:**
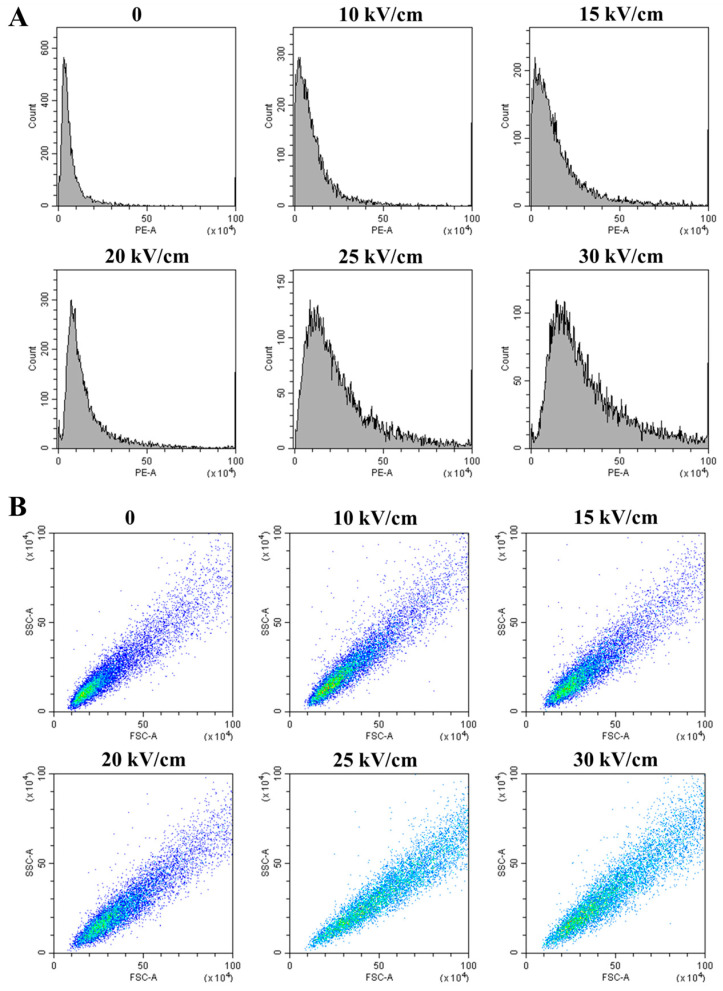
Flow cytometry analysis of *A. aceti* after PEF treatment: (**A**) Histograms of propidium iodide (PI) fluorescence intensity (PE-A), indicating membrane permeabilization. (**B**) Scatter plots of forward scatter (FSC-A) versus side scatter (SSC-A), reflecting changes in cell size and granularity. The color gradient represents the density of events (cells), ranging from blue (low density) to cyan (high density).

To quantify the loss of membrane integrity, flow cytometry with the fluorescent dye propidium iodide (PI) was employed. PI only penetrates cells with compromised membranes, making its fluorescence a direct indicator of membrane damage [28]. The fluorescence intensity histograms (Figure 3A) show a clear rightward shift in the cell population with increasing EFI, indicating a growing proportion of PI-positive cells. This was quantitatively confirmed by the mean fluorescence intensity (PE-A), which increased more than 4-fold from 1.99 × 10^5^ in the control group to 10.14 × 10^5^ at 30 kV/cm (Table 2). This dose-dependent increase in PI uptake provides quantitative proof of severe membrane permeabilization.

Furthermore, light scatter signals revealed changes in cell size (forward scatter, FSC) and internal complexity (side scatter, SSC). The scatter plots (Figure 3B) and quantitative data (Table 2) show that both mean FSC and SSC values increased significantly (*p* < 0.05) with rising EFI, particularly above 20 kV/cm. The increase in FSC suggests cell swelling, likely due to osmotic shock following the loss of membrane permeability control [31]. The concurrent increase in SSC implies a rise in internal granularity, which could result from the aggregation of intracellular macromolecules or the disorganization of internal structures following membrane failure [37]. These flow cytometry results not only quantify the membrane damage but also hint at the subsequent catastrophic events occurring within the cell.

#### 3.2.3. PEF-Induced Alterations in Membrane Fatty Acid Composition

**Figure 4:**
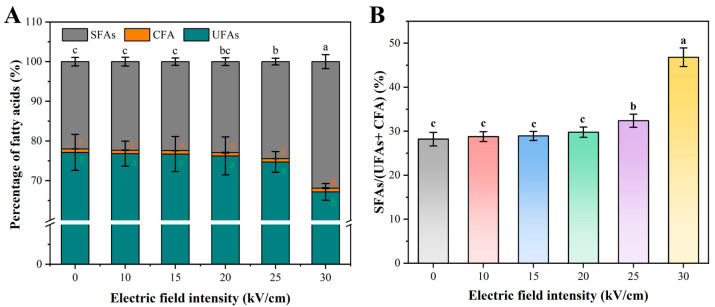
Flow cytometry analysis of *A. aceti* after PEF treatment: (**A**) Histograms of propidium iodide (PI) fluorescence intensity (PE-A), indicating membrane permeabilization. (**B**) Scatter plots of forward scatter (FSC-A) versus side scatter (SSC-A), reflecting changes in cell size and granularity. Different lowercase letters indicate significant differences between groups (*p* < 0.05).

The fatty acid profile is a key determinant of membrane fluidity, which influences the membrane’s ability to withstand stress and repair damage [30]. The membrane fatty acid composition of *A. aceti* was analyzed by GC-MS to investigate potential biochemical alterations induced by PEF. The native membrane of *A. aceti* is dominated by unsaturated fatty acids (UFAs), with oleic acid (C18: 1ω9) being the most abundant component (71.26%), and palmitic acid (C16: 0) and stearic acid (C18: 0) being the main saturated fatty acids (SFAs) (Table 3). PEF treatment induced significant changes in the fatty acid profile, particularly at high field strengths (Figure 4A). While no significant changes were observed up to 20 kV/cm, treatment at 25 and 30 kV/cm caused a significant decrease in the total UFA content (from 77.12% to 67.19%) and a corresponding significant increase in the total SFA content (from 22.00% to 31.88%) (*p* < 0.05). Consequently, the SFAs/(UFAs + CFA) ratio increased sharply from ~29% to 46.80% at 30 kV/cm (Figure 4B), indicating a substantial decrease in membrane fluidity. This shift is likely not an adaptive response but rather a direct consequence of PEF-induced damage, such as the oxidative cleavage of double bonds in UFAs by reactive oxygen species (a topic explored in Section 3.5) [38]. This conclusion is strengthened when contrasted with the adaptive mechanisms of *Acetobacter* spp. under other environmental stresses. For instance, Niu et al. reported that when *Acetobacter* sp. was exposed to increasing ethanol concentrations, the cells responded by increasing the proportion of unsaturated fatty acids to enhance membrane fluidity [28]. This adaptive fluidization helps maintain normal physiological functions under ethanol toxicity. In stark contrast, our results show the opposite effect: PEF treatment induced a significant decrease in membrane fluidity (rigidification). This opposing trend strongly supports the interpretation that the observed fatty acid alteration is not a protective strategy but rather a hallmark of catastrophic, non-recoverable oxidative damage. Thus, this PEF-induced rigidification compromises the membrane’s ability to self-repair pores created during electroporation, increasing the likelihood of irreversible damage. This provides a biochemical explanation for the sharp increase in inactivation efficiency above the critical EFI threshold.

### 3.3. PEF-Induced Leakage of Intracellular Components and Disruption of Internal Structure

The irreversible damage to the cell membrane, as demonstrated in the previous section, inevitably compromises its barrier function, leading to the leakage of intracellular components and the collapse of cellular homeostasis [39]. This section quantifies the efflux of cytoplasmic materials and visualizes the resulting internal disorganization to further elucidate the lethal effects of PEF on *A. aceti*.

#### 3.3.1. Intracellular Material Leakage

**Figure 5:**
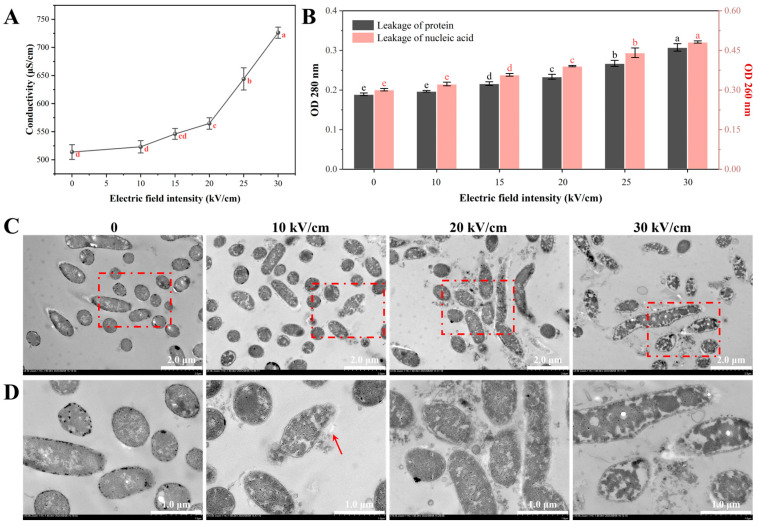
PEF-induced leakage of intracellular components and disruption of internal ultrastructure in *A. aceti*: (**A**) Changes in the electrical conductivity of the cell suspension. (**B**) Absorbance of the supernatant at 280 nm (protein leakage) and 260 nm (nucleic acid leakage). (**C**) Transmission electron microscopy (TEM) images showing an overview of cellular damage at different electric field intensities (Scale bar = 2 µm, ×4000 magnification). (**D**) Magnified TEM images showing details of ultrastructural changes (Scale bar = 1 µm, ×10,000 magnification, corresponding to the red-boxed areas in (**C**)). Red arrows indicate areas of membrane damage and structural disruption. Different lowercase letters indicate significant differences between groups (*p* < 0.05).

The leakage of ions and other charged small molecules was assessed by measuring the electrical conductivity of the cell suspension. The conductivity increased significantly with rising electric field intensity (EFI), with a sharp acceleration observed around the 20 kV/cm threshold (Figure 5A). At 30 kV/cm, the conductivity reached 726.3 µS/cm, a 41.4% increase compared to the control. The increased conductivity is a direct result of the efflux of intracellular ions (e.g., K^+^, Mg^2+^) and charged metabolites through the pores formed by electroporation [40].

Beyond small molecules, the leakage of macromolecules such as proteins and nucleic acids signifies irreversible cellular damage. The absorbance of the supernatant at 280 nm (OD_280_, for proteins) and 260 nm (OD_260_, for nucleic acids) was measured to quantify this leakage. Both OD_280_ and OD_260_ values showed a dose-dependent increase with EFI, with a significant rise observed at and above 20 kV/cm (Figure 5B). The loss of these essential biomolecules, which are central to cellular metabolism and genetic integrity, is a direct indicator of cell death [27]. These quantitative results strongly corroborate the membrane damage observed by SEM and flow cytometry (Section 3.2), confirming that PEF treatment breaches the cell’s physical barrier, leading to a fatal loss of critical cellular components.

#### 3.3.2. Internal Structural Damage Visualized by TEM

Transmission electron microscopy (TEM) was employed to directly examine the PEF-induced alterations in the internal ultrastructure of *A. aceti*. Control cells exhibited a well-defined cellular architecture, characterized by a distinct boundary between the cell wall and membrane, along with a dense and uniformly distributed cytoplasm, indicative of preserved cellular integrity (Figure 5C,D). At a low EFI of 10 kV/cm, most cells remained intact, although minor membrane blurring was occasionally visible in magnified views (red arrow). Notably, at 20 kV/cm, significant internal disorganization became evident. The cytoplasm appeared non-uniform, with the formation of low-density, vacuole-like regions, providing visual evidence of intracellular material loss [33]. At the highest EFI of 30 kV/cm, the cells suffered catastrophic damage. Severe cytoplasmic condensation and the formation of large, empty voids were observed, indicating massive leakage of cellular contents. Crucially, the cell envelope was completely compromised, showing signs of lysis and disintegration (red arrow), and the cell outline became indistinct. These observations of cytoplasmic coagulation and membrane rupture are consistent with TEM studies on other PEF-treated bacteria, such as *Bacillus cereus* and *E. coli*, where irreversible membrane rupture and leakage of cellular contents were also reported [32,33].

In summary, the TEM results provide direct visual proof of the lethal cascade initiated by PEF. They vividly illustrate the progression from an intact cell to one suffering from cytoplasmic leakage, internal chaos, and eventual lysis. These ultrastructural observations perfectly align with the quantitative leakage data, solidifying the conclusion that PEF inactivates *A. aceti* by destroying membrane integrity, leading to a fatal efflux of essential intracellular materials.

### 3.4. Inactivation of Acid-Producing Enzymes by PEF

**Figure 6:**
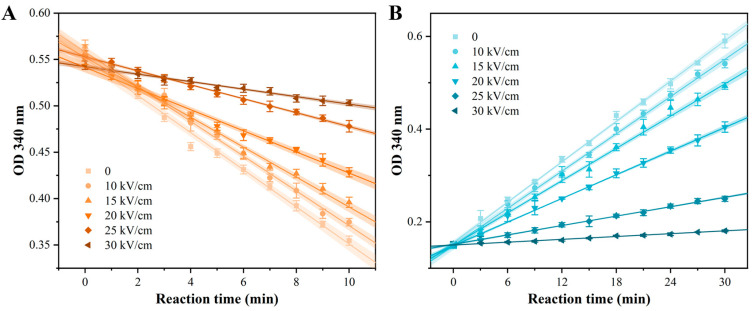
Effect of PEF treatment on the relative activity of acid-producing enzymes in *A. aceti*. The relative activity of (**A**) alcohol dehydrogenase (ADH) and (**B**) aldehyde dehydrogenase (ALDH) was monitored by the change in absorbance at 340 nm due to NADH consumption or generation, respectively. Lines represent linear fits, and shaded areas represent 95% confidence intervals.

The spoilage potential of *A. aceti* in wine stems from its ability to oxidize ethanol to acetic acid, a process catalyzed by two key intracellular enzymes: alcohol dehydrogenase (ADH) and aldehyde dehydrogenase (ALDH) [28]. To determine if PEF treatment can neutralize this spoilage capability at a biochemical level, the relative activities of ADH and ALDH were assessed. As shown in Figure 6, enzyme activities were monitored by the change in OD_340_ reflecting NADH concentration. The decreasing slope for ADH (Figure 6A) and increasing slope for ALDH (Figure 6B) represent their respective catalytic rates. A clear, dose-dependent flattening of both reaction curves was observed with increasing EFI, visually indicating significant enzyme inhibition. Quantitative analysis based on the linear fits (R^2^ > 0.98) of these curves confirmed the extent of inactivation (Table 4). The relative activity of ADH, represented by the absolute slope value, decreased by 80.0% after treatment at 30 kV/cm compared to the control. ALDH was even more sensitive, with its relative activity plummeting by 93.1% under the same conditions. These results demonstrated that PEF treatment effectively inactivated the core enzymes responsible for acetic acid production in *A. aceti*.

The mechanism of PEF-induced enzyme inactivation is primarily attributed to the disruption of protein conformation [41]. Strong electric fields can disrupt the non-covalent bonds (e.g., hydrogen bonds, hydrophobic interactions) that maintain the precise three-dimensional structure of proteins. This leads to unfolding and conformational changes, particularly in secondary (e.g., loss of α-helices) and tertiary structures, which ultimately distort the active site and abolish catalytic function [42]. Such effects have been documented for various enzymes and proteins. Therefore, the observed inactivation of ADH and ALDH is a direct consequence of PEF-induced structural damage to these enzyme proteins. This finding is crucial, as it reveals that PEF not only kills the bacterial cells but also dismantles their spoilage machinery, providing profound support for its application in wine preservation.

### 3.5. PEF-Induced Oxidative Damage and Energy Metabolic Collapse

**Figure 7:**
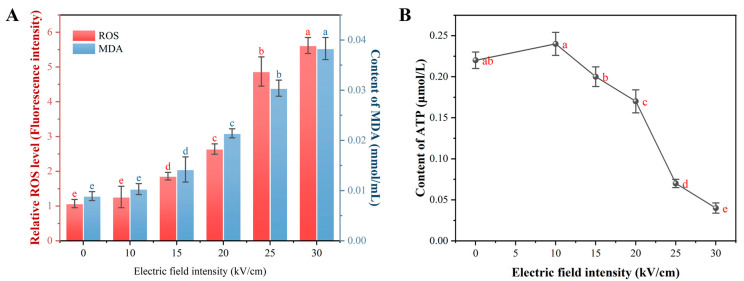
Effect of PEF treatment on oxidative stress and energy metabolism in *A. aceti*: (**A**) Intracellular reactive oxygen species (ROS) levels and malondialdehyde (MDA) content. (**B**) Intracellular ATP content. Different letters above the bars or data points indicate significant differences (*p* < 0.05).

Beyond direct electroporation, electrochemical effects of PEF, such as the generation of reactive oxygen species (ROS), can contribute significantly to its bactericidal action [20]. This section investigates the extent of PEF-induced oxidative stress and its impact on the energy metabolism of *A. aceti*. As shown in Figure 7A, PEF treatment led to a sharp, dose-dependent increase in intracellular ROS levels and malondialdehyde (MDA). This indicates that PEF triggered a chain reaction of lipid peroxidation. This oxidative attack on membrane lipids directly explains the decrease in unsaturated fatty acids (Section 3.2.3) and contributes synergistically to the physical membrane damage and permeabilization observed previously (Section 3.2.1 and Section 3.2.2). The generation of ROS, particularly hydroxyl radicals (·OH), during PEF treatment has been confirmed in other studies, underscoring its role in microbial inactivation [38,43,44].

Energy metabolism, reflected by intracellular ATP levels, was also severely disrupted by PEF treatment (Figure 7B). An initial, slight increase in ATP at 10 kV/cm suggests a transient stress response, where the cell attempts to fuel repair mechanisms. However, at EFIs of 15 kV/cm and above, ATP levels plummeted, indicating a catastrophic failure of energy homeostasis. This ATP depletion can be attributed to two main factors: (1) the massive energy expenditure by membrane-bound ATPases (e.g., H^+^-ATPase) in a futile attempt to restore ion gradients across a leaky membrane, and (2) the direct damage to enzymes and structures involved in the respiratory chain, thereby crippling ATP synthesis [45]. The collapse of the energy supply system cripples the cell’s ability to perform essential functions and repair damage, accelerating its death.

Collectively, the inactivation of *A. aceti* by PEF is a multi-faceted process. While electroporation-driven physical membrane damage is the primary event, the synergistic effects of PEF-induced oxidative stress and the subsequent collapse of energy metabolism are crucial contributors to irreversible cell death.

### 3.6. Comprehensive Analysis of the Inactivation Mechanism by Clustered Heatmap

**Figure 8:**
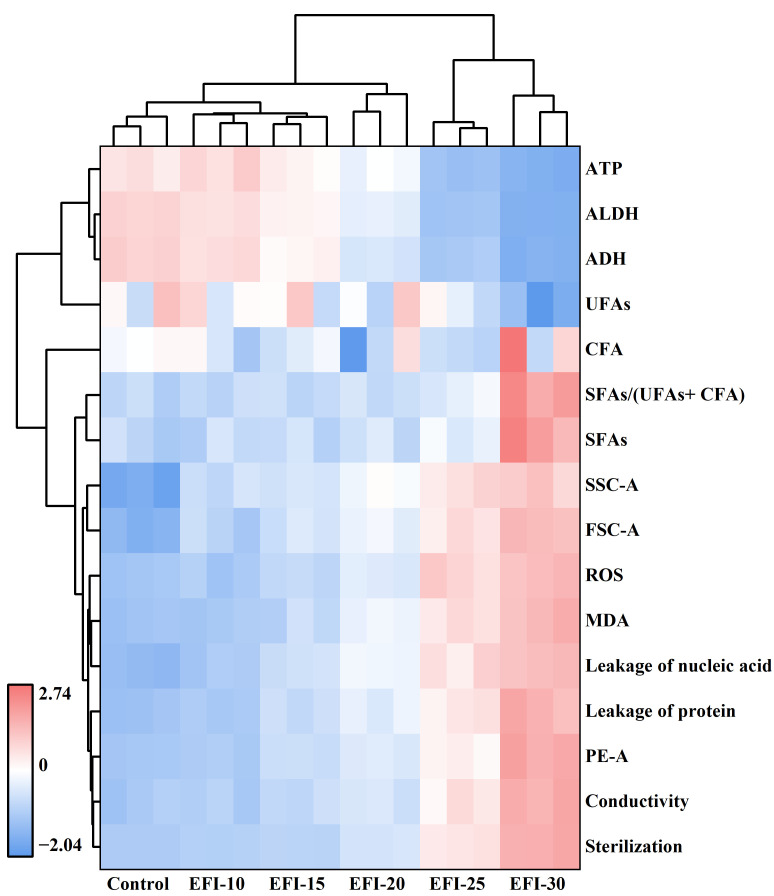
Clustered heatmap analysis of physiological and biochemical indicators in *A. aceti* following PEF treatment. The analysis visualizes the correlation between different electric field intensities (columns) and various cellular response indicators (rows).

To provide an integrated overview of the PEF inactivation mechanism, a clustered heatmap analysis was performed on all measured parameters across different EFIs (Figure 8). This multivariate approach visually synthesizes the complex cellular responses to PEF stress [46].

The column-wise hierarchical clustering clearly segregated the treatment conditions into three distinct groups: (i) the low-impact group (Control, 10, and 15 kV/cm), (ii) the transitional threshold (20 kV/cm), (iii) the high-damage group (25 and 30 kV/cm). This grouping visually corroborates the threshold-dependent nature of PEF inactivation, with the 20 kV/cm treatment marking the critical point where catastrophic failure begins, consistent with the Weibull model’s findings. The row-wise clustering categorized all measured parameters into two major super-clusters, revealing their functional relationships. The first cluster, “viability-associated indicators”, including ATP, ALDH, ADH, and UFAs, showed a strong negative correlation with EFI, confirming the suppression of vital functions. The second cluster, “damage-associated indicators”, grouped together indicators of increased membrane rigidity, oxidative stress (ROS, MDA), and structural collapse (leakage, conductivity, and cell swelling). Notably, these damage indicators cluster tightly with the final inactivation log reduction (“Sterilization”), demonstrating their direct contribution to cell death.

In summary, the heatmap provides a panoramic view confirming that PEF inactivates *A. aceti* through a synergistic, multi-target process. It illustrates that exceeding a critical EFI threshold triggers a fatal cascade, where primary membrane damage initiates comprehensive metabolic collapse, oxidative failure, and functional enzyme destruction, collectively ensuring irreversible cell death.

## 4. Conclusions

Collectively, this study provides a comprehensive elucidation of the inactivation mechanism of the wine spoilage bacterium *A. aceti* by PEF treatment as a standalone technology. Moving beyond the general confirmation of electroporation, the results systematically demonstrated that PEF inactivates *A. aceti* through a threshold-dependent and multi-target process. While electric field intensity was confirmed as the paramount parameter, the core lethal event was identified as irreversible electroporation of the cell membrane. This primary injury initiated a fatal cascade of secondary events, including the loss of cellular homeostasis through massive component leakage, a catastrophic collapse of energy metabolism, and severe oxidative damage. The synergistic action ensures rapid and efficient cell death. By demonstrating the effective inactivation of not only the bacterial cells but also their key acid-producing enzymes (ADH and ALDH), this research provides a strong theoretical basis for PEF as a potential alternative to traditional control methods. It suggests that PEF treatment can simultaneously eliminate the spoilage microorganism and its immediate spoilage potential. These insights, derived from a simulated wine matrix, provide a strong theoretical basis for developing PEF into an efficient control strategy.

However, translating these findings into industrial practice requires careful consideration of several critical challenges. It is crucial that future research validate these fundamental findings in real wine matrices, where components such as ethanol, sugars, and organic acids will alter the medium’s conductivity and thus PEF’s energy efficiency and inactivation efficacy. Furthermore, the potential negative impacts of the PEF treatment itself must be thoroughly evaluated. For instance, electrochemical reactions occurring at the electrode surface or the generation of free radicals could lead to the oxidation of sensitive phenolic compounds, potentially affecting wine color, bitterness, and aging capacity. Similarly, any minor thermal effects or oxidative stress could degrade delicate volatile aroma compounds, compromising the wine’s bouquet. Therefore, it is essential to find a delicate balance: PEF parameters must be intense enough to achieve the desired microbial inactivation but gentle enough to preserve the wine’s intricate chemical and sensory profile. This will likely involve exploring shorter pulse durations and advanced chamber designs with highly efficient cooling systems.

## Figures and Tables

**Table 1:** Weibull model fitting parameters for the inactivation of *Acetobacter aceti* under different PEF treatments.

PEF Parameters	Weibull Fitting Parameters				
	a	b	R^2^	RMSE	SSE
EFI	21.64 ± 0.21	3.48 ± 0.12	0.9856	0.1464	0.0858
PF	15.73 ± 2.99	0.38 ± 0.03	0.9967	0.0651	0.0169
PW	4.23 ± 0.15	1.32 ± 0.07	0.9732	0.1924	0.1481
RT	1.77 ± 0.08	0.73 ± 0.02	0.9897	0.1176	0.0554

**Table 2:** Quantitative flow cytometry parameters of *A. aceti* after PEF treatment at different electric field intensities.

Electric Field Intensity (kV/cm)	Mean FSC-A (×10^5^)	Mean SSC-A (×10^5^)	Mean PE-A (×10^5^)
0	6.21 ± 0.33 e	5.82 ± 0.37 d	1.99 ± 0.07 e
10	8.37 ± 0.84 d	9.30 ± 0.48 c	2.25 ± 0.15 e
15	9.49 ± 0.46 d	9.68 ± 0.20 c	3.49 ± 0.11 d
20	10.52 ± 0.41 c	11.18 ± 0.39 b	4.31 ± 0.14 c
25	12.86 ± 0.60 b	12.98 ± 0.58 a	6.39 ± 0.29 b
30	14.91 ± 0.27 a	13.86 ± 0.61 a	10.14 ± 0.40 a

Different lowercase letters indicate significant differences between groups (*p* < 0.05).

**Table 3:** Detailed membrane fatty acid composition of *A. aceti* after PEF treatment at different electric field intensities.

Fatty Acids (%)	Electric Field Intensity (kV/cm)					
	0	10	15	20	25	30
Saturated fatty acids, SFAs						
C14: 0	2.45 ± 0.82 c	2.55 ± 0.46 c	2.67 ± 0.38 c	3.14 ± 0.58 bc	3.89 ± 0.57 b	4.59 ± 0.13 a
C16: 0	12.63 ± 1.01 b	12.75 ± 1.24 b	12.88 ± 0.99 b	12.97 ± 0.53 b	13.09 ± 0.67 b	16.61 ± 0.20 a
C18: 0	5.96 ± 0.44 b	5.92 ± 0.48 b	5.95 ± 0.59 b	5.99 ± 0.55 b	6.38 ± 0.34 b	9.09 ± 0.82 a
C20: 0	0.96 ± 0.21 bc	1.12 ± 0.09 b	0.94 ± 0.11 bc	0.84 ± 0.07 c	1.10 ± 0.07 b	1.59 ± 0.11 a
Total of SFAs	22.00 ± 1.07 c	22.34 ± 1.11 c	22.44 ± 0.94 c	22.94 ± 0.97 bc	24.46 ± 0.84 b	31.88 ± 1.77 a
Unsaturated fatty acids, UFAs						
C16: 1ω9	5.42 ± 0.35 a	5.21 ± 0.40 a	5.00 ± 0.17 a	4.93 ± 0.33 a	4.55 ± 0.24 b	4.35 ± 0.14 b
C18:1ω9	71.26 ± 4.52 a	71.18 ± 2.00 a	71.30 ± 4.17 a	70.90 ± 2.66 a	69.82 ± 1.17 a	62.51 ± 2.12 b
C20:1ω9	0.44 ± 0.02 a	0.43 ± 0.03 a	0.41 ± 0.12 a	0.41 ± 0.10 a	0.35 ± 0.07 a	0.33 ± 0.01 a
Total of UFAs	77.12 ± 3.51 a	76.82 ± 3.13 a	76.71 ± 4.43 a	76.24 ± 4.78 a	74.72 ± 2.61 a	67.19 ± 2.11 b
Cyclic fatty acid, CFA						
C19: cyclo	0.88 ± 0.01 a	0.84 ± 0.05 a	0.85 ± 0.02 a	0.82 ± 0.10 a	0.82 ± 0.01 a	0.93 ± 0.11 a
SFAs/(UFAs + CFA)	28.21 ± 1.54 c	28.77 ± 1.13 c	28.93 ± 1.03 c	29.77 ± 1.17 c	32.38 ± 1.49 b	46.80 ± 2.12 a

Different lowercase letters indicate significant differences between groups (*p* < 0.05).

**Table 4:** Linear fitting parameters for the reaction kinetics of ADH and ALDH relative activity in *A. aceti* after PEF treatment at different electric field intensities.

	**EFI (kV/cm)**	**Slope**	**Error**	**Intercept**	**Error**	**R^2^**
ADH	0	−0.0200	0.0004	0.5511	0.0025	0.9917
	10	−0.0187	0.0005	0.5582	0.0029	0.9952
	15	−0.0161	0.0005	0.5523	0.0031	0.9921
	20	−0.0114	0.0004	0.5414	0.0022	0.9874
	25	−0.0075	0.0004	0.5528	0.0023	0.9957
	30	−0.0040	0.0004	0.5423	0.0021	0.9807
	**EFI (kV/cm)**	**Slope**	**Error**	**Intercept**	**Error**	**R^2^**
ALDH	0	0.0145	0.0002	0.1553	0.0022	0.9986
	10	0.0134	0.0003	0.1487	0.0043	0.99695
	15	0.0116	0.0002	0.1496	0.0035	0.9979
	20	0.0085	0.0002	0.1484	0.0033	0.99758
	25	0.0034	0.0002	0.1516	0.0034	0.99692
	30	0.0010	0.0000	0.1500	0.0010	0.99289

## Data Availability

The original contributions presented in this study are included in the article. Further inquiries can be directed to the corresponding authors.
